# Activated T cell-derived exosomal PD-1 attenuates PD-L1-induced immune dysfunction in triple-negative breast cancer

**DOI:** 10.1038/s41388-021-01896-1

**Published:** 2021-06-25

**Authors:** Yufan Qiu, Yi Yang, Riyao Yang, Chunxiao Liu, Jung-Mao Hsu, Zhou Jiang, Linlin Sun, Yongkun Wei, Chia-Wei Li, Dihua Yu, Jin Zhang, Mien-Chie Hung

**Affiliations:** 1 3rd Department of Breast Cancer Prevention, Treatment and Research Center; Key Laboratory of Breast Cancer Prevention and Therapy (Ministry of Education); Tianjin’s Clinical Research Center for Cancer; Key Laboratory of Cancer Prevention and Therapy, Tianjin; National Clinical Research Center for Cancer, Tianjin Medical University Cancer Institute and Hospital, Tianjin, PR China; 2 Department of Molecular and Cellular Oncology, The University of Texas MD Anderson Cancer Center, Houston, TX USA; 3 Tianjin Medical University, Tianjin, PR China; 4 Institution of Pathology and Southwest Cancer Center, Southwest Hospital, Third Military Medical University (Army Medical University), Chongqing, PR China; 5 Graduate Institute of Biomedical Sciences and Research Center for Molecular Medicine, China Medical University, Taichung, 406 Taiwan; 6 Tianjin Key Laboratory of Lung Cancer Metastasis and Tumor Microenvironment, Lung Cancer Institute, Tianjin Medical University General Hospital, Tianjin, PR China; 7 Institute of Biomedical Sciences, Academia Sinica, Taipei, Taiwan; 8 Department of Biotechnology, Asia University, Taichung, 413 Taiwan

**Keywords:** Breast cancer, Cell death, Immunotherapy

## Abstract

Programmed cell death 1 (PD-1) is widely expressed in tumor-infiltrating lymphocytes (TILs) of triple-negative breast cancer (TNBC). As a dominant inhibitory immune checkpoint (ICP) receptor, cell surface PD-1 is well-known to transduce negative signaling of effector T cell activity during cell–cell contact. However, despite its well-documented inhibitory effects, higher PD-1 expression in TILs is significantly associated with longer survival in TNBC patients. This phenomenon raises an interesting question whether PD-1 harbors positive activity to enhance anti-tumor immunity. Here, we show that PD-1 is secreted in an exosomal form by activated T cells and can remotely interact with either cell surface or exosomal programmed death-ligand 1 (PD-L1), induce PD-L1 internalization via clathrin-mediated endocytosis, and thereby prevent subsequent cellular PD-L1: PD-1 interaction, restoring tumor surveillance through attenuating PD-L1-induced suppression of tumor-specific cytotoxic T cell activity. Our results, through revealing an anti-PD-L1 function of exosomal PD-1, provide a positive role to enhance cytotoxic T cell activity and a potential therapeutic strategy of modifying the exosome surface with membrane-bound inhibitory ICP receptors to attenuate the suppressive tumor immune microenvironment.

## Introduction

Triple-negative breast cancer (TNBC) is a group of highly heterogeneous tumors with diverse biological behavior and clinical outcomes. Research has shown that 33% of non-inflammatory TNBC cases had an immunomodulatory signature, and the frequency of cases with this signature was highest (48%) in the basal-like 1 subtype [1]. Among TNBC tumors, 21.9% comprise more than 50% tumor-infiltrating lymphocytes (TILs), and the median lymphocyte infiltration percentage is ~20% among all cases [2]. A high proportion of TILs, proposed as a marker of the immunomodulatory signature, is strongly associated with a favorable prognosis despite the ununified classification of TNBC genomic subtypes [3, 4].

Programmed cell death 1 (PD-1) is one of the co-inhibitory immune checkpoint (ICP) receptors induced upon T cell activation and widely expressed (70.3%) in TILs [5]. Through transducing negative signaling of effector T cell activity by the interaction with programmed death-ligand 1 (PD-L1, CD274), PD-1 serves as a mediator for tumor cells to survive by escaping T cell killing [6, 7]. However, interestingly, PD-1 expression is significantly associated with longer disease-free survival and overall survival in patients with TNBC [5]. Consistent results have shown that high *PDCD1* gene transcription levels within TNBC tumors and high numbers of PD-1-positive immune infiltrates are associated with significantly increased disease-free survival [8]. Together, these findings raised an interesting question: whether PD-1 on T cells might somehow associate with possible functions in restricting the immune evasion of tumor cells aside from the conventional immunosuppressive activity?

The tumor immune microenvironment is a milieu containing complex systemic networks between diverse components including cell-to-cell communication mediated by multiple types of cellular transport. Exosomes are single-membrane, secreted extracellular vesicles (EVs) 30–200 nm in diameter that originate from plasma and endosomes [9]. Depending on their cells of origin, exosomes are enriched with a wide variety of contents, and are widely involved in intercellular communications within tumors [10, 11].

Exosomes derived from T lymphocytes are involved in the regulation of immune reaction [12, 13]. Depending on the cell classification and status, the functions of exosomes secreted by tumor-associated T cells can be diverse and complicated [14]. Here in this study, we revealed that PD-1 secreted in an exosomal form protects against the anti-tumor immune dysfunction induced by PD-L1 in TNBC and therefore offers a potential application for surface modification of therapeutic exosomes.

## Results

### Exosomes carrying membrane-bound PD-1 are released by activated T cells

**Fig. 1:**
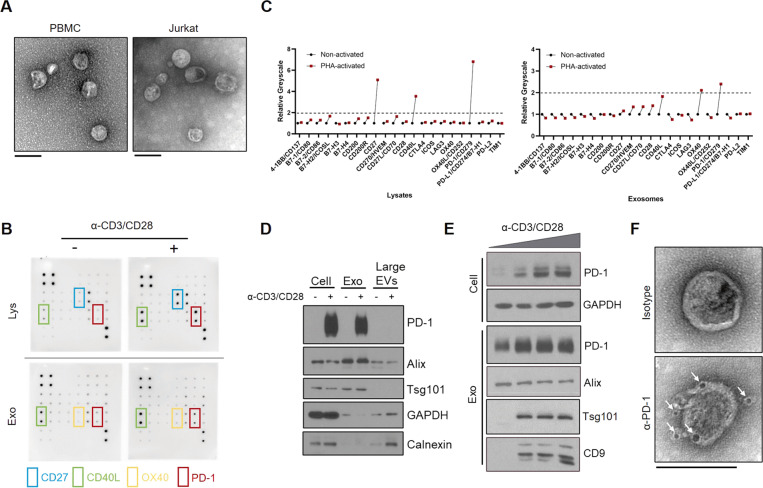
Characterization of T cell-released exosomal PD-1. **A** TEM images of exosomes derived from activated PBMC-T (left) and Jurkat-T cells (right). Scale bar, 100 nm. **B** Immune checkpoint array of non-activated (left) and CD3/CD28 activator-activated (right) Jurkat cellular lysates (Lys) and exosomes (Exo). Molecule layout map is shown in Supplementary Fig. 1. **C** Greyscale value quantification of immunoblot spots listed in **B**. Tim-3 was excluded due to undetectable expression level. PHA phytohemagglutinin. **D** Immunoblot of PD-1 expression in cells, exosomes, and large EVs in Jurkat cells. Exosomes were collected from the culture supernatants of non-activated or CD3/CD28 activator-stimulated Jurkat cells in 72 h. Exosomes were normalized by identical protein quantity. Alix and Tsg101, exosome markers. GAPDH and Calnexin, cell plasma markers. **E** Immunoblot of cellular and exosomal PD-1 and exosome markers (Alix, Tsg101, and CD9) with increasing anti-CD3/CD28 T cell activator concentration in PBMC-T cells in 72 h. **F** TEM images of PD-1 distribution on the exosome membrane. White arrows, anti-PD-1 antibody-conjugated 5 nm gold nanoparticles. Scale bar, 100 nm.

To study the biological implications of Exo-PD-1 using the proper model, we first isolated exosomes derived from peripheral blood mononuclear cells (PBMCs)-derived T cells and Jurkat-T cells. As shown in transmission electron microscopy (TEM) images (Fig. 1A), exosomes released by PBMC-T and Jurkat-T cells shared similar morphological characteristics, with a typical size range of 50–100 nm in diameter. Then, to analyze the expression pattern of immune checkpoints carried by T cell exosomes in a non-biased approach, we used an ICP array to test the exosomes secreted from T-cell-receptor (TCR)-non-activated and activated T cells (Fig. 1B and Supplementary Fig. 1). According to the comprehensive interpretation of immunoblot images and corresponding greyscale values (Fig. 1C), PD-1 appeared to have the most significant change among the tested exosomal ICPs. Next, by isolating diverse samples of cells, exosomes (small EVs) and large EVs, we validated the result of the immune checkpoint array that exosomal PD-1 was released by T cells upon TCR stimulation, and also confirmed that EV-related PD-1 was specifically carried by exosomes instead of other larger EVs (Fig. 1D). We also validated the expression of Exo-PD-1 in PBMC-T cells, and the result showed that Exo-PD-1 secretion was enhanced by the increasing intensity of TCR stimulation (Fig. 1E).

Next, by labeling activated T cell-derived exosomes with gold nanoparticle-conjugated anti-PD-1 antibody and visualized using TEM scanning, PD-1 was located on the outer surface of exosomes, indicated by black particles around exosomes (lower panel, Fig. 1F). In comparison, no exosome-attached particles can be seen in isotype control despite treating with an equal amount of gold nanoparticle-conjugated antibody (upper panel, Fig. 1F). With these results, we revealed that T lymphocytes release exosomal membrane-bound PD-1 upon activation.

### Exo-PD-1 enhances T cell-mediated killing of cancer cells

**Fig. 2:**
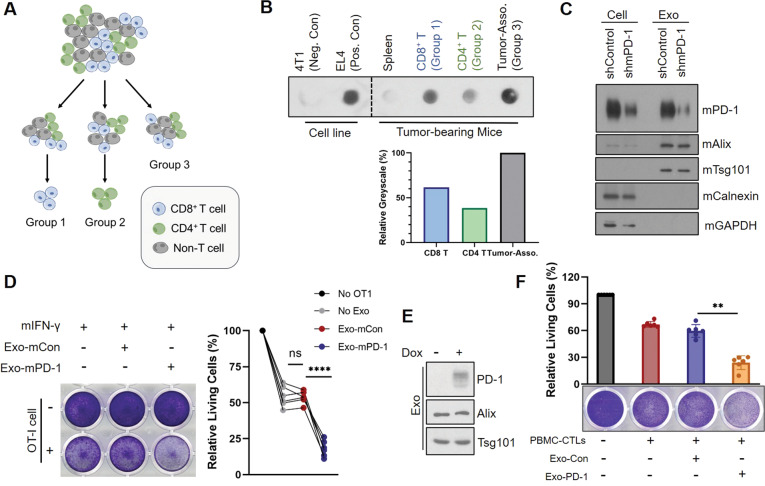
Exo-PD-1 enhances the killing capacity of cytotoxic T lymphocytes in vitro. **A** Illustration showing the grouping of exosome-donor cells from which exosomes used in **B** were derived. Single cells isolated from 4T1 tumors were equally divided into three groups. CD8^+^ T cells were isolated from Group 1 (blue cells), CD4^+^ T cells were isolated from Group 2 (green cells), and no isolation was performed in Group 3 to mimic original cellular microenvironment within the tumors. **B** Immunoblots (upper) and relative greyscale value quantification of exosomal PD-1 expression in 4T1 tumor-bearing splenic cells, CD8^+^ and CD4^+^ tumor-infiltrating T lymphocytes (Group 1 and Group 2, respectively), and tumor-associated cells (Group 3). The value of Group 3 was set as 100% for comparison. Exosomes from 4T1 cells were used as the negative control, and exosomes from EL4 cells as the positive control to validate anti-mPD-1 antibody specificity. **C** Immunoblot of cellular and exosomal mPD-1 in wild-type and mPD-1 knockdown mouse EL4-T cell lines. Exosomes from EL4-shmPD-1 were used as Exo-mCon and exosomes from EL4-shControl as Exo-mPD-1 in the following experiments. mAlix, mTsg101, mouse exosome markers. mCalnexin, mGAPDH, mouse cell plasma markers. **D** Representative crystal violet staining images (left) and absorption quantification (right) of remaining living PY8119-OVA cells after co-culture with OT-I cells in the absence or presence of Exo-mCon or Exo-mPD-1 for 2 days. Each curve represents one independent experiment (*n* = 6). ns no statistical significance. ****P < 0.00005. **E** Immunoblot of cellular and exosomal PD-1 expression in doxycycline-inducible Jurkat-PD-1 cells. **F** Crystal violet staining images (lower) and absorption quantification (upper) of remaining living BT549-PD-L1^−/−^ cells after PBMC-T cell killing in the presence of Exo-Con or Exo-PD-1 for 2 days (*n* = 6). **P < 0.005.

Since we have validated that exosomal PD-1 was released by activated T lymphocytes, it was also important to investigate which T cell population was responsible for producing Exo-PD-1 during tumor-induced immunoreaction. Therefore, we isolated exosomes from 4T1 tumor-bearing splenic cells, tumor-associated cells, tumor-infiltrating CD8^+^ T cells and CD4^+^ T cells (Fig. 2A). Comparing to the splenic cells, Exo-PD-1 was mostly released by tumor-associated cells (Fig. 2B). Further quantitative analysis showed that the total Exo-PD-1 mostly came from T lymphocytes (both CD8^+^ and CD4^+^) infiltrated within the tumors (indicated as the relative identical greyscale values between Group 3 and the sum of Group 1 and Group 2). In addition, the expression level of CD8^+^ T cell-derived Exo-PD-1 was higher than Exo-PD-1 from CD4^+^ T cells, yet generally speaking, tumor-infiltrating CD8^+^ and CD4^+^ T lymphocytes both contributed to the production of Exo-PD-1 within tumor microenvironment. This result indicates that antigen-activated TILs that migrated from lymphatic tissues are the main producers of exosomal PD-1 in anti-tumor immunity.

To determine the biological function of Exo-PD-1, it was necessary to identify its role during the confrontation between cytotoxic T lymphocytes (CTLs) and tumor cells. Ovalbumin (OVA)-overexpressing PY8119, a TNBC cell line from C57BL/6J mice, was generated as a model for an in vitro OT-I cell-killing assay. CTLs isolated from OT-I mice can be activated by OVA_257–264_ peptide and specifically recognize OVA antigens expressed on the surface of PY8119-OVA tumor cells. First, OVA and surface PD-L1 expression of PY8119-OVA cells were validated (Supplementary Fig. 2A, B). Next, we used EL4, a T lymphoma cell line from C57BL/6J mice, as a donor of mouse exosomal PD-1 (Exo-mPD-1, shown as shControl in Fig. 2C) and generated its PD-1-knockdown clone as a control (Exo-mCon, shown as shmPD-1 in Fig. 2C). Then, we co-cultured mouse IFN-γ-pretreated PY8119-OVA cells with activated OT-I CTLs in the absence or presence of Exo-mCon or Exo-mPD-1, respectively. As shown, the CTL killing effect was significantly enhanced by Exo-mPD-1 treatment (Fig. 2D). Also, to exclude the impacts of EL4 exosomes on the cytotoxicity of OT-I CTLs and the vitality of tumor cells, we tested the proliferation and cytotoxic cytokine production of OT-I cells, including IFN-γ, granzyme B, perforin, and TNF-α (Supplementary Fig. 3A, B), and no significance changes were found. We also observed no significant changes on the proliferation and vitality of exosome-treated PY8119-OVA cells (Supplementary Fig. 4). These results suggest that Exo-PD-1 may play a defensive role against tumor-cell surface PD-L1-induced CTL dysfunction and consequently enhance the cytotoxicity of tumor-specific CTLs.

Moreover, studies have shown that PD-L1 expressed on T lymphocytes also harbors immune-suppressive functions [15, 16]. On the basis of these findings, we validated the PD-L1 expression in PMBC-T cells (Supplementary Fig. 5) and performed an in vitro T cell-killing assay by co-culturing these effector T cells with PD-L1-knockout BT549 (human TNBC cell line) cells to exclude the interference from tumor cell-derived PD-L1. In this assay, doxycycline-inducible Jurkat-PD-1 cells were used as donors for Exo-Con/Exo-PD-1 (Fig. 2E). The significant reduction of living tumor cells (Fig. 2F) indicated that Exo-PD-1 also thwarts immune suppression caused by the interaction between PD-1 and PD-L1 in effector T cells. Altogether, these in vitro data showed that Exo-PD-1 enhances the cytotoxic activity of effector T cells against tumor cells in the tumor immune microenvironment.

### Exo-PD-1 competitively occupies PD-L1 and attenuates subsequent T cell dysfunction

**Fig. 3:**
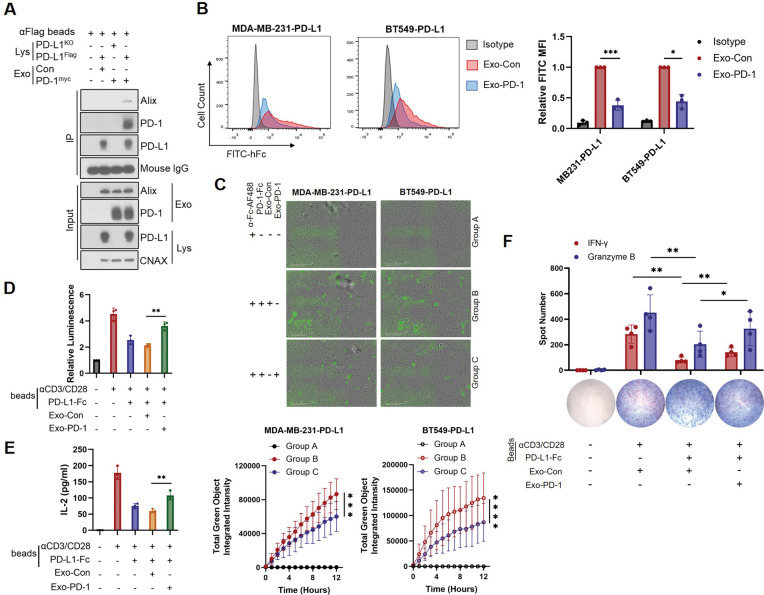
Exo-PD-1 binds cell surface PD-L1. **A** Immunoprecipitation and western blots showing the interaction between exosomal PD-1 and cellular PD-L1. **B** Representative flow cytometry images (left) and quantitative analysis (right) of cell surface interacted rhPD-1-Fc level in Exo-Con or Exo-PD-1-treated PD-L1-overexpression breast cancer cells (*n* = 3). *P < 0.05, ***P < 0.0005. **C** Time-lapse evaluation and quantification of the dynamic interaction between Alexa Fluo^TM^ 488-labeled rhPD-1-Fc (green) and surface PD-L1 in breast cancer cells in the presence of Exo-Con or Exo-PD-1 treatment for 12 h (*n* = 3). ***P < 0.0005, ****P < 0.00005. **D** Normalized luminescence of Exo-Con or Exo-PD-1 pretreated stimulatory (anti-CD3/CD28/IgG) or inhibitory (anti-CD3/CD28/PD-L1) bead-stimulated Jurkat-PD-1-NFAT-Luciferase reporter cells for 24 h (*n* = 3). **E** Quantification of IL-2 secreted by Jurkat-PD-1 cells stimulated with stimulatory (anti-CD3/CD28/IgG) or inhibitory (anti-CD3/CD28/PD-L1) beads pre-incubated with Exo-Con or Exo-PD-1 (*n* = 3). **P < 0.005. **F** ImmunoSpot microscopy images and spot quantification of stimulatory (anti-CD3/CD28/IgG) or inhibitory (anti-CD3/CD28/PD-L1) bead-stimulated PBMC-T cell-released IFN-γ (red) and granzyme B (blue) particles in the presence of Exo-Con or Exo-PD-1 (*n* = 4). *P < 0.05, **P < 0.005.

Next, to validate that Exo-PD-1 attenuates tumor cell-induced T cell dysfunction via the interaction with cell-surface PD-L1, we needed to confirm the binding between Exo-PD-1 and cellular PD-L1 first. Therefore, we used an immunoprecipitation-western blot assay to show that a bead-PD-L1 complex could only bind Exo-PD-1 and not Exo-Con. In turn, Exo-PD-1 could only bind PD-L1-conjugated beads and not PD-L1-absent beads (Fig. 3A). This direct evidence indicates that PD-1 carried by exosomes can interact with cellular PD-L1 in vitro.

On the basis of this result, we next asked whether the interaction between Exo-PD-1 and PD-L1 was capable of preventing the binding of PD-L1 to non-exosomal PD-1. Compared to Exo-Con, Exo-PD-1 significantly attenuated the binding of recombinant human PD-1-Fc (rhPD-1-Fc) to the cell surface (Fig. 3B). Then we performed an assay using the time-lapse live-cell culture system to better observe the binding process of rhPD-1-Fc visually. By dynamically measuring green fluorescence-conjugated PD-1-Fc levels, we showed that the binding efficiency of rhPD-1-Fc was significantly lower in Exo-PD-1-treated tumor cells than in Exo-Con-treated cells (Fig. 3C). These results indicate a competitive space-occupying effect of Exo-PD-1, which prevents PD-L1 from binding to non-exosomal exogenous PD-1 molecules.

Next, we further investigated Exo-PD-1 function against the PD-L1 molecule during T cell activation. To measuring the alteration of activation level in T cells, we generated NFAT-Luciferase-transfected Jurkat-PD-1 cells and treated with anti-CD3/CD28/IgG (stimulatory) or anti-CD3/CD28/PD-L1 (inhibitory) conjugated beads that were pre-incubated with Exo-Con/PD-1. As NFAT is a downstream factor activated upon TCR ligation, and IL-2 is a main reactive cytokine secreted during T cell activation, significantly higher levels of luminescence (Fig. 3D) and secreted IL-2 (Fig. 3E) suggested that Exo-PD-1 could block PD-L1 to weaken subsequent immunosuppression. Thus, we then validated this conclusion in PBMC-derived activated effector T cells with a consistent result that IFN-γ and granzyme B levels inhibited by PD-L1-conjugated beads were significantly rescued by Exo-PD-1 but not by Exo-Con (Fig. 3F), suggesting Exo-PD-1 harbors the function of immune restoration against PD-L1-induced immunosuppression. Altogether, these results provide strong evidence of exosomal PD-1 remotely occupying tumor-cell surface PD-L1 binding sites and preventing the binding of PD-L1 to T-cell surface PD-1.

### Exo-PD-1 induces PD-L1 internalization via clathrin-mediated endocytosis

**Fig. 4:**
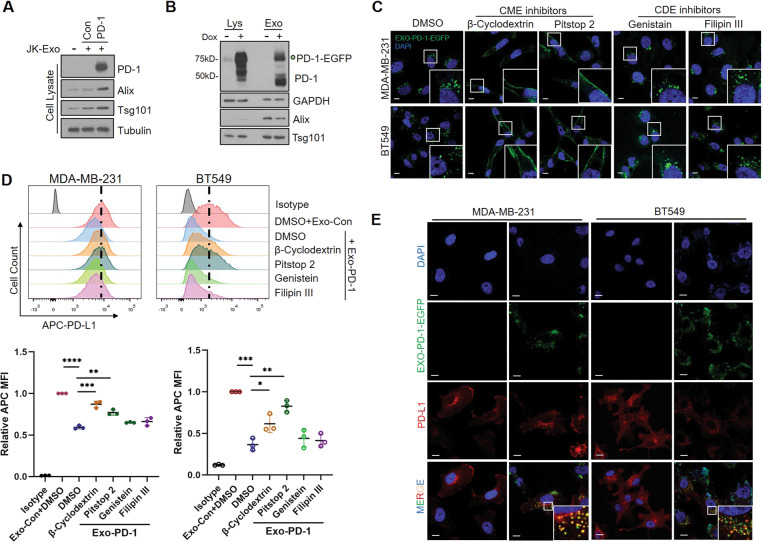
Exo-PD-1 induces cell surface PD-L1 internalization. **A** Immunoblot of cellular PD-1 and exosome uptake in BT549-PD-L1 cells with Exo-Con or Exo-PD-1 treatment. **B** Immunoblot of cellular and exosomal PD-1-EGFP expression in doxycycline-inducible Jurkat-PD-1-EGFP cell line. **C** Confocal microscopy images of Exo-PD-1-EGFP (green) uptake with or without treatments with multiple endocytosis inhibitors. CME clathrin-mediated endocytosis, CDE caveolae-dependent endocytosis. Scale bar, 10 μm. **D** Representative flow cytometry images (upper) and quantitative analysis (lower) of breast cancer cell surface PD-L1 levels with or without Exo-Con or Exo-PD-1 treatments combined with diverse endocytosis inhibitors (*n* = 3). *P < 0.05, **P < 0.005, ***P < 0.0005, ****P < 0.00005. **E** Confocal immunofluorescence microscopy images of Exo-PD-1-EGFP (green): cellular PD-L1 (red) internalization and co-localization in PD-L1-overexpression TNBC cells. Scale bar, 10 μm.

Furthermore, we investigated the consequent outcome of Exo-PD-1 binding to PD-L1 on the surface of tumor cells. Interestingly, we found that Exo-PD-1 could be taken up by recipient PD-L1-overexpressing tumor cells more efficiently than Exo-Con (Fig. 4A) by cell-to-exosome incubation. This result indicated a possible exosomal transportation approach mediated by an Exo-PD-1-to-PD-L1 interaction. Previous publications have shown that the mechanism of exosome uptake differs among diverse recipient cell types [17, 18]. Therefore, by treating with Exo-PD-1-EGFP (Fig. 4B) and recording the green florescent using confocal imaging, we showed that Exo-PD-1 uptake was blocked and trapped on the cell surface by clathrin-mediated endocytosis (CME) inhibitors, whereas little effect was observed with caveolae-dependent endocytosis (CDE) inhibitor treatment (Fig. 4C). This result suggests that CME acts as the major mechanism by which Exo-PD-1 enters the TNBC cells.

Moreover, a significant reduction of surface PD-L1 in tumor cells after Exo-PD-1 treatment was observed, which could be rescued by CME inhibitors but not CDE inhibitors (Fig. 4D), suggesting a possible clathrin-mediated downregulation of PD-L1 induced by Exo-PD-1. Confocal imaging also showed that Exo-PD-1 co-localized with endocytosed PD-L1 in the cytoplasm (Fig. 4E), providing straightforward evidence of the endocytosis induced by Exo-PD-1-to-PD-L1 interaction. Altogether, these results demonstrate that Exo-PD-1 from T cells not only binds PD-L1 on the cell surface but also induces CME of PD-L1, thereby reducing the amount of surface PD-L1 available during subsequent cancer-to-T cell direct interaction.

### Exo-PD-1 forms exosomal clusters with Exo-PD-L1 and rescues T cell activity

**Fig. 5:**
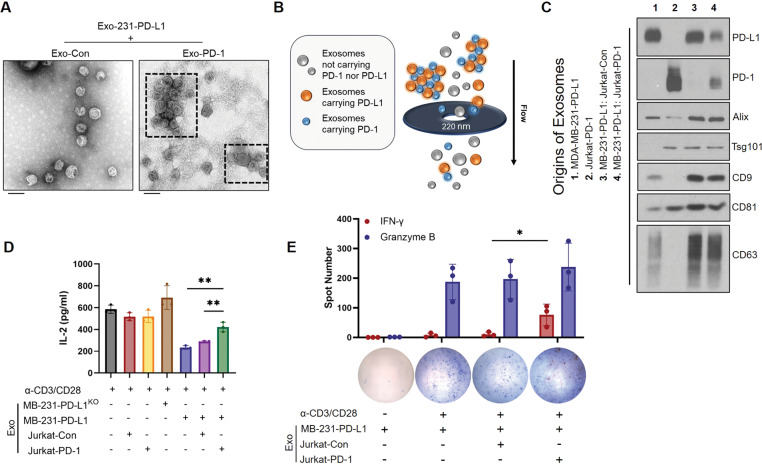
Exo-PD-1 neutralizes Exo-PD-L1-induced T cell dysfunction by forming exosomal clusters. **A** TEM images of mixtures of exosomes derived from MDA-MB-231-PD-L1 with Exo-Con (left) and Exo-PD-1 (right). Clusters of exosomes are shown within dashed frames. Scale bar, 100 nm. **B** Illustration showing that large Exo-PD-L1: Exo-PD-1 clusters are impeded and excluded by 0.22 μm filter pores. **C** Immunoblot of exosomal PD-1, PD-L1, and exosome markers (Alix, Tsg101, CD9, CD81, CD63) of exosomes isolated from the culture supernatants of indicated four groups (left). **D** Quantification of IL-2 secreted by activated PBMC-T cells treated with diverse groups of exosomes as indicated (*n* = 3). **P < 0.005. **E** ELISPOT images and spot quantification of activated PBMC-T cell-released IFN-γ (red) and granzyme B (blue) particles with diverse exosomal treatments as indicated (*n* = 3). *P < 0.05.

A previous study has shown that exosomes of different cell origins can physically interact with one another to attenuate consequent signaling [19]. On the basis of these findings, we hypothesized that Exo-PD-1 released by T lymphocytes might be able to interact with exosomal PD-L1 (Exo-PD-L1) released by tumor cells. To validate our hypothesis, we first incubated exosomes isolated from PD-L1-overexpressing MDA-MB-231 cells (Exo-231-PD-L1) with Exo-Con or Exo-PD-1 in vitro, respectively. By TEM imaging, clusters of exosomes could be observed in Exo-231-PD-L1: Exo-PD-1 mixture but not in Exo-231-PD-L1: Exo-Con mixture (Fig. 5A).

Next, to validate whether the clusters of exosomes are formed by Exo-PD-L1: Exo-PD-1 interactions in the extracellular matrix, we mimicked the microenvironmental cell interactions by culturing cells as four groups: MB-231-PD-L1 cells only (Group 1), Jurkat-PD-1 cells only (Group 2), MB-231-PD-L1: Jurkat-Con cells co-culture (Group 3), and MB-231-PD-L1: Jurkat-PD-1 cells co-culture (Group 4). Then, exosomes from each group were filtered through 0.22 μm membranes, which are used for separating freeing exosomes from exosomal aggregates and large EVs in conventional exosome purification, and isolated by ultracentrifugation before testing the exosomal PD-L1/PD-1 expression levels by immunoblotting. Theoretically, exosome clusters larger than 0.22 μm are not able to pass through the filter pores. Therefore, the components carried by these clusters of exosomes will be reduced (Fig. 5B). As expected, the total amounts of Exo-PD-L1 and Exo-PD-1 in the MB-231-PD-L1: Jurkat-PD-1 co-culture group significantly decreased, indicating that Exo-PD-1 forms clusters with Exo-PD-L1 in extracellular matrix (Fig. 5C).

Next, we asked whether the formation of Exo-PD-L1: Exo-PD-1 clusters attenuates Exo-PD-L1-induced T cell dysfunction. To answer this question, we isolated exosomes from seven groups of cell culture supernatants, as indicated in Fig. 5D. To mimic the microenvironment, we kept the Exo-PD-L1: Exo-PD-1 clusters in the co-culture system by not filtering the supernatants before ultracentrifugation. As represented by IL-2 levels, the suppression of PBMC-T cell activity was significantly rescued by exosomes from the MB-231-PD-L1: Jurkat-PD-1 co-culture group, which contained Exo-PD-L1: Exo-PD-1 clusters (Fig. 5D). Furthermore, the IFN-γ level of PBMC-effector T lymphocytes was significantly restored by Exo-PD-L1: Exo-PD-1 interaction (Fig. 5E). Altogether, these findings demonstrate that Exo-PD-1 interacts and forms clusters with Exo-PD-L1 and consequently attenuates T cell suppression induced by Exo-PD-L1.

### Exo-PD-1 inhibits tumor growth and enhances anti-tumor immunity

**Fig. 6:**
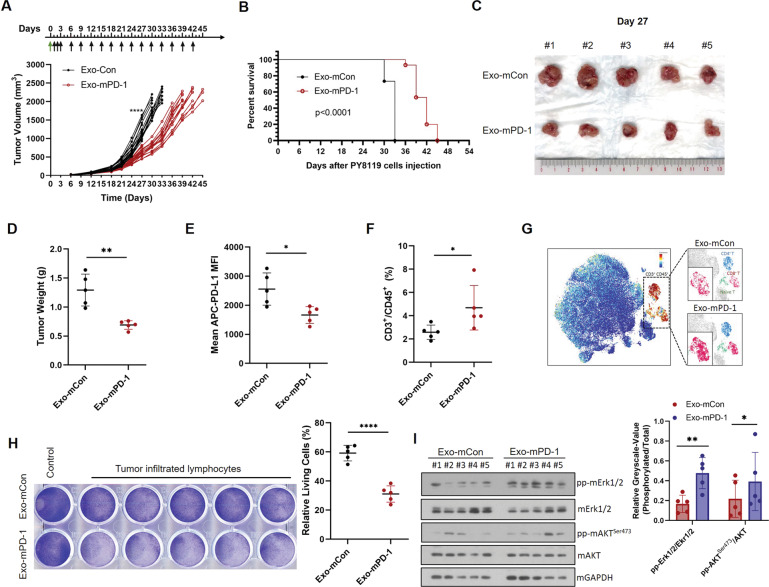
Exo-PD-1 inhibits tumor growth in a TNBC animal model. **A** The growth curve of orthotopic PY8119 tumors in C57BL/6J mice following Exo-Con or Exo-PD-1 treatment. Black arrows, timepoints of exosome treatments. Green arrow, seeding of PY8119 cells. Each curve represents data recorded from one mouse. 20 mice per group in day 0 to day 27, and 15 mice per group after day 27. ****P < 0.00005. **B** Kaplan–Meier survival curve for tumor-bearing mice during exosome treatment. Sizes (**C**) and weights (**D**) of tumors in Exo-mCon- and Exo-mPD-1-treatment groups. 5 tumors in each group were excised randomly on day 27. **P < 0.005. **E** Flow cytometry analysis of surface PD-L1 level in PY8119 tumor cells on day 27 (*n* = 5). *P < 0.05. **F** Flow cytometry analysis of T cell percentage within PY8119 tumor-infiltrating immune cells (*n* = 5). *P < 0.05. **G** CyTOF mass cytometry analysis of T cell infiltration in PY8119 tumors. Orange, CD3^+^ cell population (total T lymphocytes). Blue, CD4^+^ T cell population. Cherry, CD8^+^ T cell population. Green, naïve T cell population. **H** Crystal violet staining images and absorption quantification of remaining living PY8119 cells after co-culturing with activated PY8119 tumor-infiltrating T cells in the presence of Exo-mCon or Exo-mPD-1, respectively (*n* = 5). ****P < 0.00005. **I** Immunoblots (left) and greyscale value quantification (right) of representative TCR downstream signals, phosoho-Erk1/2 and phospho-AKT^Ser473^, in TILs isolated from exosome-treated PY8119 tumors. Each lane represents sample isolated from one tumor in **C**. *P < 0.05, **P < 0.005.

To validate the anti-tumor function of Exo-PD-1 in vivo, Exo-mCon or Exo-mPD-1 isolated from mouse T cells was then injected intraperitoneally to PY8119 tumor-bearing mice according to the study design. As shown, Exo-mPD-1 significantly slowed tumor growth (Fig. 6A) and increased survival duration (Fig. 6B) in tumor-bearing mice. Compared to the Exo-mCon treatment group, tumors were significantly smaller (Fig. 6C) and lighter (Fig. 6D) in the Exo-mPD-1 group.

Next, to investigate the alterations in both tumor cells and T cells during Exo-PD-1 treatment, we analyzed the cell distribution and surface markers in the tumor immune microenvironment. We observed significantly decreased cell surface PD-L1 levels (Fig. 6E) and increased T cell infiltration rates in the Exo-PD-1 treatment group (Fig. 6F). We also found that the CD8^+^ T cell population was significantly increased by Exo-PD-1 treatment (indicated by the higher intensity of clusters with cherry color), suggesting an enhanced anti-tumor cytotoxicity of TILs (Fig. 6G).

To further validate whether CTLs play an essential role in the exosomal PD-1-induced anti-tumor effect, we isolated and expanded tumor-infiltrating T cells for in vitro cell-killing assay. Consistent with the in vivo results, Exo-mPD-1 treatment enhanced the T cell-mediated killing of PY8119 cells (Fig. 6H). In addition, we tested the expression level of phosphor-Erk1/2 and phosphor-AKT^Ser473^, which are downstream signals of TCR pathway, in TILs isolated from the tumors of these two groups. And the stronger signals indicated enhanced TCR activation in TILs of Exo-mPD-1-treated tumors (Fig. 6I). Altogether, these results demonstrate that the anti-tumor function of exosomal PD-1 is mediated by a decreased PD-L1 burden within the tumor and the enhanced cell-killing capacity of tumor-infiltrating CTLs.

## Discussion

**Fig. 7:**
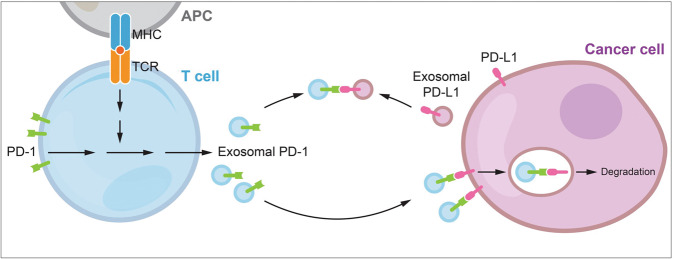
A proposed model of PD-L1 blockade by activated T cell-derived exosomal PD-1 particles. T cells activated upon antigen recognition release exosomal PD-1 to restrict surface and exosomal PD-L1-induced inhibition. Exosomal PD-1 can either block surface PD-L1 loci and induce consequent tumor PD-L1 internalization via clathrin-mediated endocytosis, or neutralize exosomal PD-L1 and prevent its binding to T cell surface PD-1. Altogether, exosomal PD-1 helps to maintain T cell cytotoxicity against tumor cells.

Immune checkpoints play essential regulatory roles in tumor immune surveillance. Among them, PD-1 is broadly expressed in activated tumor-associated effector T lymphocytes and currently regarded as a dominant inhibitory regulator in anti-tumor adaptive immunity [20, 21]. However, the immunosuppressive role of PD-1 seems to be conflicting with its clinical implication. Studies of TNBC and other cancer types have shown a positive correlation between PD-1 expression in TILs and better clinical outcome, especially in PD-1^+^ CD8^+^ T cell population [22–24]. This conflict is currently partially explained by TIL activation indicated by PD-1 expression, but it has not been reported whether in any circumstance that PD-1 molecules play a positive role in anti-tumor immunity. Here in this study, we first discovered the biofunction of exosomal PD-1 derived from activated T cells in defending against PD-L1-induced tumor evasion (Fig. 7). In addition to PD-1, high proportions of TILs expressing other ICP receptors that transfer inhibitory signaling for T cell activation and survival also represent an independent favorable prognostic factor in hormone receptor-negative breast cancer [25, 26]. Whether these markers of T cell apoptosis and exhaustion are carried by T cell-derived exosomes, and whether they harbor similar immune function with exosomal PD-1 in tumor immunity deserve further investigation.

As a promising but relatively immature area of cancer research, numerous studies on exosomes have emerged [27, 28]. Inconsistent results of immunoregulatory functions are observed in different groups of T lymphocytes [14]. For instance, within the tumor environment, CD8^+^ T cells prevent tumor progression by causing EV-mediated depletion of mesenchymal tumor stromal cells [29]; whereas the cytotoxicity of activated CD8^+^ T cells can be inhibited by exosomes released from exhausted CD8^+^ T cells [30]. In our current study, besides exosomal PD-1, we also observed significant changes in exosomal OX40 and CD40L (Fig. 1B, C). It has been previously shown that non-tumor-cell-derived exosomal OX40 is negatively correlated with apoptosis of CD8^+^ T lymphocytes, indicating a potential protective role of exosomal OX40 with unknown mechanism [31]. In addition, exosomal CD40L was reported being involved in efficient induction of anti-tumor immunity mediated by T lymphocytes [32]. The secretion of T cell exosomes is highly increased upon T cell activation through enhanced polarization [33, 34], yet, the specific intracellular regulation and the distribution of these activation-upregulated molecules on exosomes is still largely unknown. Validating whether these exosomal components are co-expressed in single exosome particle or selected precisely into different exosome populations might help explain the diverse functions of T cell-derived exosomes.

As a cell surface transmembrane protein, PD-L1 was first regarded to cause immune suppression merely during direct cell-to-cell contact. However, several recent studies consistently showed that exosomal PD-L1 secreted by tumor cells remotely impairs regular immune surveillance by triggering the PD-1 inhibitory pathway in T cells, which consequently contributes to tumor evasion [35, 36]. Nevertheless, little research has been done on the correlation between serum exosomal PD-1 levels and prognosis in cancer patients, and the predictive value of serum exosomal PD-1 as a biomarker still remains controversial in different types of cancer [37, 38]. Compared to serum exosomal PD-L1, the serum exosomal PD-1 levels might not be able to represent the real status of the tumor immune microenvironment. Unlike the continuous secretion of cancerous exosomes, the transfer of T cell exosomes is intensely triggered and strictly directional from T lymphocytes to recipient cells through rapid immune synapse during activation [34, 39, 40]. Consequently, detecting exosomal PD-1 alone in blood and body fluids can be difficult and unreliable. Nonetheless, given its biofunction against PD-L1, exosomal PD-1, in cooperation with other exosomal ICP receptors, could still possibly be applied as one of a group of comprehensive tumor prognostic markers in future investigations with clinical samples.

Currently, EVs are being studied as promising carriers for drugs and nucleic acids [41, 42]. As a nanoscale delivery system, exosomes possess unique characteristics based on their native biological membrane structure [43]. Hence, the modification of the exosomal membrane is as essential as that of internal cargos. In light of our findings of exosomal PD-1, we also provide a potential strategy of cell surface modification based on therapeutic tumor-targeting exosomes. Inhibitory ICP receptors expressed by effector T cells are currently well-documented as immune suppressors. However, based on our current study, it would be rational to discover novel approaches to either transfer these surface inhibitory ICPs into releasable exosomal forms, or directly stimulate the polarization and secretion of T cell exosomal-ICP inhibitory receptors. Furthermore, in combination with chemotherapy agents or nucleic acid cargos, exosomes loaded with surface inhibitory ICP receptors, such as PD-1, Tim-3, and LAG-3, might be able to attenuate the immune-suppressive microenvironment extracellularly while simultaneously attacking tumor cells intracellularly.

## Material and methods

### Cell lines and antibodies

All wild-type cell lines were purchased from the ATCC (Manassas, VA, USA), independently validated by short tandem repeat DNA fingerprinting at The University of Texas MD Anderson Cancer Center, and negative for mycoplasma infection. PBMCs from three donors were purchased from STEMCELL Technologies (Vancouver, Canada). Culture conditions for cell lines are shown in Supplementary Table S1. Antibodies applied in this study are shown in Supplementary Table S2.

### Plasmids

Mouse PD-1 knockdown plasmids were obtained from PDCD1 MISSION shRNA Bacterial Glycerol Stock purchased from Sigma-Aldrich (Product type SHCLNG-NM_008798, St. Louis, MO, USA). The generation of modified doxycycline-inducible human PD-1-myc/EGFP/NFAT-Luc and PD-L1-Flag constructs and corresponding stable overexpression transfectants has been described previously, and these materials are stored in our lab [44, 45].

### Generation of stable cells by lentiviral infection

The expression or knockdown constructs were transfected into HEK 293T cells following a protocol described previously [46]. Detailed description is shown in the supplementary information.

### Isolation and purification of exosomes

Exosomes were collected from cell culture supernatants and purified by sequential ultracentrifugation as described previously [47]. Detailed description is shown in the supplementary information. In this study, alix, tsg101, CD9, CD81, and CD63 were used as markers of exosomes in immunoblot assays. Calnexin was used as a negative control of exosomes according to the recommendation of The International Society for EVs [48].

### Transmission electron microscopy

TEM of exosome samples was performed at the High-Resolution Electron Microscopy Facility at MD Anderson Cancer Center. Detailed description is shown in the supplementary information.

### Immunoblot assays

Detailed description of immunoblot assays, including immune checkpoint array, western blot assay, dot blot assay and immunoprecipitation assay is shown in the supplementary information.

### Flow cytometry

The cell surface staining was performed following BioLegend’s protocol. Detailed description of cell surface staining and PD-1-Fc binding assay is shown in the supplementary information.

### Time-lapse live-cell imaging

Adherent MDA-MB-231-PD-L1 and BT549-PD-L1 cells were incubated with exogeneous Exo-Con or Exo-PD-1 for 1 h in a cell incubator before rhPD-1-Fc protein and anti-human IgG-Fc Alexa Fluro^TM^ 488 (BioLegend, San Diego, CA, USA) were added to the co-culture system. The fluorescence intensity was then recorded by the real-time IncuCyte live-cell analysis system (Essen BioScience, Ann Arbor, MI, USA).

### IL-2 ELISA assay

In Fig. 3E, Jurkat-PD-1 cells (2 × 10^5^/200 μl/well) were seeded in 96-well plates in the presence of pretreated beads and cultured for 48 h. In Fig. 5D, Jurkat-PD-1 cells (2 × 10^5^/200 μl/well) were seeded in 96-well plates in the presence of anti-CD3/CD28 activator (5 μl/ml) and exosomes and cultured for 48 h. Culture supernatants were then collected and the IL-2 concentration was measured using Human IL-2 ELISA MAX Deluxe (BioLegend).

### NFAT-luciferase reporter assay

Jurkat-PD-1-NFAT-Luciferase-transfected cells (1 × 10^5^/100 μl/well) were seeded in 96-well plates in the presence of pretreated beads and cultured for 24 h. Luminescence values were acquired using the Bright-Glo™ Luciferase Assay System (Promega, Madison, WI, USA).

### Enzymatic ELISPOT assay

Activated PBMCs (1 × 10^5^ cells/100 μl/well) from three donors were seeded and treated as indicated for 24 h before being subjected to IFN-γ/granzyme B double-color enzymatic ELISPOT assay (ImmunoSpot^®^, Cleveland, OH, USA). Images and data were recorded at Oncology Research and Immuno-monitoring Core at MD Anderson Cancer Center.

### Immunofluorescence

For exosome uptake imaging and PD-L1: Exo-PD-1 co-internalization imaging, detailed description is shown in the supplementary information. Images were recorded and analyzed by a confocal microscope (LSM700, Carl Zeiss, Oberkochen, Germany).

### OT-I cell-killing assay

OT-I cells harvested from the spleens of C57BL/6-Tg (TcraTcrb)1100Mjb/J mice (7-week-old females) were cultured in vitro for 72 h and seeded at ratios of 5:1 to adherent PY8119-OVA cells and co-cultured for 48 h in the presence of Exo-mCon or Exo-mPD-1. The dosing of exosomes samples was calculated by protein concentration. 1 × 10^5^ PY8119 cells were treated with 30 μg exosomes in 500 μl culture media in 24-well plates. The remaining adherent cells were stained by 0.5% crystal violet solution in 25% methanol for 10 min. Stained cells were eluted with 10% glacial acetic acid (2 ml) and the optical density at 590 nm was recorded.

### PBMC-T cell-killing assay

PBMCs from three donors were used for independent and duplicated experiments. In total, data from six independent experiments were recorded. Activated PBMC-T cells were seeded at a ratio of 7:1 to adherent BT549 cells and co-cultured for 48 h in the presence of Exo-Con or Exo-PD-1 (30 μg per 1 × 10^5^ BT549 cells in 500 μl culture media). Staining and data recording were performed as described above.

### Animal studies

All experimental animals were purchased from The Jackson Laboratory (Bar Harbor, ME, USA) performed in accordance with guidelines approved by MD Anderson’s Institutional Animal Care and Use Committee (protocol tittle: IACUC Study #00001250-RN02). The experiment design is briefly described in the results and the figure legend of Fig. 2A, detailed description is shown in the supplementary information.

For in vivo exosomal treatment in Fig. 6, 40 C57BL/6 mice (6-week-old females) were randomly assigned into Exo-mCon or Exo-mPD-1 treatment group to minimize the individual variance. No significant difference of body weight between the two groups was observed. PY8119 cells (4 × 10^5^ cells) were injected into the mammary fat pads (one tumor per mouse). Exosomes were administered to mice daily by intraperitoneal injection for 3 continuous days (120 μg/mouse/treatment in 100 μl PBS) after the injection of tumor cells and once every 3 days afterward (240 μg/mouse/treatment in 100 μl PBS) until the experimental end point, natural death or tumor size reaching 2000 mm^3^. Tumor volume was calculated by using the formula π/6 × length × width × height. On day 27, five mice in each group were randomly euthanized for in vitro T cell-killing assay, immunoblots, mass cytometry, and flow cytometry analysis. Exosome treatments were given to the 15 remaining mice as designed in each group for survival analysis. No animals were excluded from the analysis in this study.

### Mass cytometry analysis

Mass cytometry analysis was performed by the MD Anderson Flow Cytometry and Cellular Imaging Core Facility. Detailed description of antibody staining and data analysis is shown in the supplementary information.

### Tumor-infiltrating T cell-killing assay

Tumor-infiltrating T cells were isolated using the EasySep™ Mouse T Cell Isolation Kit (STEMCELL Technologies) from single-cell samples prepared from exosome-treated PY8119 tumors. Cells were then added to pre-seeded adherent PY8119 cells for 48 h. Staining and data analysis were performed as described above.

### Statistical analysis

Statistical analyses were performed using GraphPad Prism 8 software. Paired Student’s *t* tests were used to compare differences between groups in Figs. 1–5. Unpaired Student’s *t* tests were used in Fig. 6. A two-tailed *P* value < 0.05 was considered statistically significant. Experiments were performed three or more times independently. Each dot or curve in the figures represents data from one independent experiment. Randomization was used in the grouping and euthanasia in animal studies.

## Supplementary information

Supplementary Information: Material and Methods

Supplementary Information: Figures and Tables
