# Supplementary material for: Activated T cell-derived exosomal PD-1 attenuates PD-L1-induced immune dysfunction in triple-negative breast cancer
Source: Oncogene. 2021 Jun 25;40(31):4992–5001. doi: 10.1038/s41388-021-01896-1 (PMC8342306; doi:10.1038/s41388-021-01896-1)
Supplement: Supplementary file 1 — Supplementary Information: Material and Methods [file 41388_2021_1896_MOESM1_ESM.docx]

Activated T cell-derived exosomal PD-1 attenuates PD-L1-induced immune dysfunction in triple-negative breast cancer

Yufan Qiu^1,2,4^, Yi Yang^5,2^, Riyao Yang^2^, Chunxiao Liu^2^, Jung-Mao Hsu^3,2^, Zhou Jiang^2^, Linlin Sun^7,2^, Yongkun Wei^2^, Chia-Wei Li^8,2^, Dihua Yu^2^, Jin Zhang*^1,4^, Mien-Chie Hung*^3,2,6^

^1^3rd Department of Breast Cancer Prevention, Treatment and Research Center; Key Laboratory of Breast Cancer Prevention and Therapy (Ministry of Education); National Clinical Research Center for Cancer, Tianjin Medical University Cancer Institute & Hospital, Tianjin Medical University, Tianjin, PR China.

^2^Department of Molecular and Cellular Oncology, The University of Texas MD Anderson Cancer Center, Houston, TX, USA.

^3^Graduate Institute of Biomedical Sciences and Research Center for Molecular Medicine, China Medical University, Taichung 406, Taiwan.

^4^Key Laboratory of Cancer Prevention and Therapy; Tianjin's Clinical Research Center for Cancer, Tianjin, PR China

^5^Institution of Pathology and Southwest Cancer Center, Southwest Hospital, Third Military Medical University (Army Medical University), Chongqing, PR China.

^6^Department of Biotechnology, Asia University, Taichung 413, Taiwan

^7^Tianjin Key Laboratory of Lung Cancer Metastasis and Tumor Microenvironment, Lung Cancer Institute, Tianjin Medical University General Hospital, Tianjin, PR China.

^8^Institute of Biomedical Sciences, Academia Sinica, Taipei, Taiwan

Supplementary information:

Material and methods

Generation of stable cells by lentiviral infection

The medium was changed at 24 h after transfection and supernatant collected after an additional 48 h. The centrifuged supernatants were filtered using 0.45 μm filters. Adherent cells (30% confluency) or suspended cells (5 × 10^5^ cells/ml) were cultured in transfected-293T-derived lentivirus-containing medium with polybrene (8 μg/ml; EMD Millipore, Burlington, MA, USA) for 24 h and subjected to puromycin selection (2 μg/ml; InvivoGen, San Diego, CA, USA) in 72 h following the infection.

Isolation and purification of exosomes

Cells were cultured for 72 h and the supernatants were collected by centrifugation at 300g for 10 min and 2 000g for 10 min to exclude cells and cell debris. Supernatants were then centrifuged at 10 000g for 30 min to enrich large extracellular vesicles. Pellets were lysed with RIPA lysis buffer for Western blot assay. The remaining solution was then passed through filters with 0.22 μm pore size (if required by the experimental design) and ultracentrifuged at 100 000g for 70 min to pellet the exosomes. The pellets were then washed in a large volume of PBS to eliminate contaminating proteins and centrifuged one last time at 100 000g for 70 min. The final pellets that contained exosomes were re-suspended in PBS or culture media.

Transmission electron microscopy

Exosome samples were placed on 100 mesh carbon-coated and formvar-coated copper grids treated with poly-l-lysine for approximately 30 min. Samples were then negatively stained with Millipore filtered aqueous 1% uranyl acetate for 1 min. For 5-nm gold nanoparticle labeling, grids were transferred on drops of diluted primary anti-PD-1 antibody overnight at 4 °C followed by incubation of diluted secondary gold antibody (1:20, Sigma-Aldrich) for 2 h at room temperature. Stains were blotted dry from the grids with filter paper, and the samples were allowed to dry before being examined in a JEM 1010 transmission electron microscope (TEM) (JEOL, Peabody, MA, USA, Inc.) at an accelerating voltage of 80 Kv. Digital images were obtained using the AMT Imaging System (Advanced Microscopy Techniques Corp.).

Immune checkpoint array

Cells or isolated exosomes were lysed using 2× lysis buffer contained in the RayBio^®^ C-Series Human Immune Checkpoint Array 1 (RayBiotech, Peachtree Corners, GA, USA). Blocking and antibody incubation were performed following the manufacturer’s instructions. Images were recorded using a ChemiDoc imaging system (Bio-Rad, Hercules, CA, USA). Greyscale-value quantification was measured by ImageJ software.

Western blot assay

Protein samples (concentration determined using the BCA Protein Assay Kit, Sigma-Aldrich) were resolved by SDS-PAGE and transferred onto methanol-activated polyvinylidene difluoride (PVDF) membranes (Millipore) at 250mA for 70 min. The membranes were blocked in 8% fat-free dry milk with 0.05% PBS-T for 1 h at room temperature, followed by staining of the primary antibodies for 16 h at 4 °C. Then, the membranes were washed and incubated with corresponding horseradish peroxidase-conjugated secondary antibodies for 1 h at room temperature. Signals of specific proteins were developed with enhanced chemiluminescence detection (Pierce Biotechnology, Rockford, IL, USA) and images were acquired by darkroom techniques.

Dot blot assay

Exosome samples in PBS were loaded on methanol-activated PVDF membranes by vacuuming. The membranes were blocked in 8% fat-free dry milk with 1% PBS-T for 1 h at room temperature, followed by additional blocking in 5% BSA with 1% PBS-T overnight at 4°C. The membranes were then stained with the primary mouse PD-1 antibody (Cell Signaling Technology, Danvers, MA, USA) for 16 h at 4°C followed by washing, incubation of secondary antibody and signal development, which are identical with Western blot assay.

Immunoprecipitation assay

MDA-MB-231 cells expressing PD-L1-Flag were lysed in PTY buffer (50 mM HEPES, 50 mM NaCl, 5 mM EDTA, 1% Triton X-100, 50 mM NaF, 10 mM Na_4_P_2_O_7_; pH to 7.4) containing protease inhibitors. Lysates were incubated with anti-FLAG^®^ M2 magnetic beads (Sigma-Aldrich) overnight at 4 °C. Beads were washed 5 times with 1× PBS buffer containing 0.005% NP-40 (PBS-NP-40) and incubated with Exo-Con or Exo-PD-1 dissolved in washing buffer for 4 h at 4 °C, followed by washing 5 times with 1 ml PBS-NP-40 buffer. Beads with proteins were eluted in 1× SDS sample buffer and subjected to Western blot assay.

Flow cytometry

**Cell surface staining.** Cells were washed in pre-cooling PBS once and suspended in 100 ml cell staining buffer (CSB; BioLegend). The cells were then incubated with fluorescence-conjugated antibody on ice for 20 min in the dark followed by washing with CSB. Data were acquired using a BD FACSCanto II flow cytometer (BD Biosciences, San Jose, CA, USA).

**PD-1-Fc binding assay.** Adherent MDA-MB-231 and BT549 cells (2 × 10^5^) were suspended in 50 μl CSB following trypsin digestion and FBS neutralization. Cells were then cultured with Exo-Con or Exo-PD-1 in 100 μl volume for 1 h on ice at 4 °C to suspend the uptake of exosomes. Then, 10 ng rhPD-1-Fc protein was added per sample and incubated for 1 h before incubating with anti-human IgG-Fc-Alexa Fluro^TM^ 488 antibody for 20 min on ice in the dark. Cells were washed, and their fluorescence values were recorded using a BD FACSCanto II flow cytometer.

Immunofluorescence

For exosome uptake imaging, adherent MDA-MB-231-PD-L1 and BT549-PD-L1 cells were treated with methyl-β-cyclodextrin, Pitstop2, genistein, or Fillipin III (Sigma-Aldrich) for 2 h before Exo-PD-1-EGFP was added for an additional 4 h. Then cells were fixed with 4% paraformaldehyde for 20 min. Nuclear staining was performed with VECTASHIELD mounting medium and DAPI (Vector Laboratories, Burlingame, CA, USA).

For PD-L1: Exo-PD-1 co-internalization imaging, adherent cells were treated with Exo-PD-1-EGFP for 8 h, then gently washed by PBS buffer and fixed with 4% paraformaldehyde for 15 min, followed by permeabilization using 0.5% Triton X-100 for 15 min. Cells were then blocked with 5% BSA-PBS-T for 1 h and stained with anti-PD-L1 extracellular domain primary antibody for 16 h at 4 °C. Next, cells were incubated with anti-rabbit Alexa Fluo^TM^ 594 secondary antibody for 1 h at room temperature. Nuclear staining was performed with VECTASHIELD mounting medium and DAPI (Vector Laboratories). Images were recorded and analyzed by a confocal microscope (LSM700, Carl Zeiss).

Animal studies

For *in-vitro* dot blot assay in Figure 2A, 4T1 cells (5 × 10^4^ cells per tumor, 2 tumors per mouse) were injected into the mammary fat pads of 10 Balb/c mice (6-week-old females). In 21 days, tumors and spleens of the tumor-bearing mice were collected and single cells were prepared using 70μm filters and RBC lysis buffer (BioLegend). Tumors were dissected and digested into single cells using the Tumor Dissociation Kit (Miltenyi Biotec, Bergisch Gladbach, Germany) and gentleMACS™ Dissociator. For each mouse, 1 × 10^8^ splenic cells were collected and cultured in RPMI1640 with 10% de-activated FBS; tumor-associated cells were collected and divided equally into three groups to minimize the variance. (Group 1-3, 1 × 10^8^ cells per group). Cells in Group 1 and Group 2 were cultured overnight to exclude the adherent cells, and suspensive cells were then collected. CD8^+^ cells in Group 1 were isolated by Mojosort Mouse CD8 T Cell Isolation Kit (BioLegend), and CD4^+^ T cells in Group 2 were isolated by Mojosort Mouse CD4 T Cell Isolation Kit (BioLegend). Cells in Group 3 were cultured without further purification to preserve the similarity of original tumor microenvironment. After culturing in-vitro for 72 h, exosomes were then isolated from merged culture media in each group and resuspended into 50 μl PBS. Exosomal mouse PD-1 level was then tested by dot blot assay with anti-mPD-1 antibody. EL4 cell-derived exosomes were used as the positive control and 4T1 cell-derived exosomes as the negative control.

Mass cytometry analysis

PY8119 tumors were digested using the Tumor Dissociation Kit (Miltenyi Biotec) and gentleMACS™ Dissociator. Before staining with metal-labeled cell-surface CyTOF monoclonal antibody cocktail (MD Anderson Cancer Center), cells were incubated with anti-mouse CD16/32 antibody (BioLegend) for 15 min to block Fc receptors. Then, cells were permeabilized by methanol, followed by incubation with intracellular antibodies. Cell samples were washed and diluted in ddH_2_O containing bead standards to approximately 1 × 10^6^ cells/ml for mass cytometry analysis performed by the MD Anderson Flow Cytometry and Cellular Imaging Core Facility. All mass cytometry files were normalized and gated by FlowJo software to exclude dead cells, debris, doublets, and beads. An unsupervised Phenograph clustering analysis in the R CyTOF kit package was performed to automatically identify immune profiling.
