# Supplementary material for: Activated T cell-derived exosomal PD-1 attenuates PD-L1-induced immune dysfunction in triple-negative breast cancer
Source: Oncogene. 2021 Jun 25;40(31):4992–5001. doi: 10.1038/s41388-021-01896-1 (PMC8342306; doi:10.1038/s41388-021-01896-1)
Supplement: Supplementary file 2 — Supplementary Information: Figures and Tables [file 41388_2021_1896_MOESM2_ESM.docx]

Activated T cell-derived exosomal PD-1 attenuates PD-L1-induced immune dysfunction in triple-negative breast cancer

Yufan Qiu^1,2,4^, Yi Yang^5,2^, Riyao Yang^2^, Chunxiao Liu^2^, Jung-Mao Hsu^3,2^, Zhou Jiang^2^, Linlin Sun^7,2^, Yongkun Wei^2^, Chia-Wei Li^8,2^, Dihua Yu^2^, Jin Zhang*^1,4^, Mien-Chie Hung*^3,2,6^

^1^3rd Department of Breast Cancer Prevention, Treatment and Research Center; Key Laboratory of Breast Cancer Prevention and Therapy (Ministry of Education); National Clinical Research Center for Cancer, Tianjin Medical University Cancer Institute & Hospital, Tianjin Medical University, Tianjin, PR China.

^2^Department of Molecular and Cellular Oncology, The University of Texas MD Anderson Cancer Center, Houston, TX, USA.

^3^Graduate Institute of Biomedical Sciences and Research Center for Molecular Medicine, China Medical University, Taichung 406, Taiwan.

^4^Key Laboratory of Cancer Prevention and Therapy; Tianjin's Clinical Research Center for Cancer, Tianjin, PR China

^5^Institution of Pathology and Southwest Cancer Center, Southwest Hospital, Third Military Medical University (Army Medical University), Chongqing, PR China.

^6^Department of Biotechnology, Asia University, Taichung 413, Taiwan

^7^Tianjin Key Laboratory of Lung Cancer Metastasis and Tumor Microenvironment, Lung Cancer Institute, Tianjin Medical University General Hospital, Tianjin, PR China.

^8^Institute of Biomedical Sciences, Academia Sinica, Taipei, Taiwan

Supplementary Data

Supplementary Figure 1


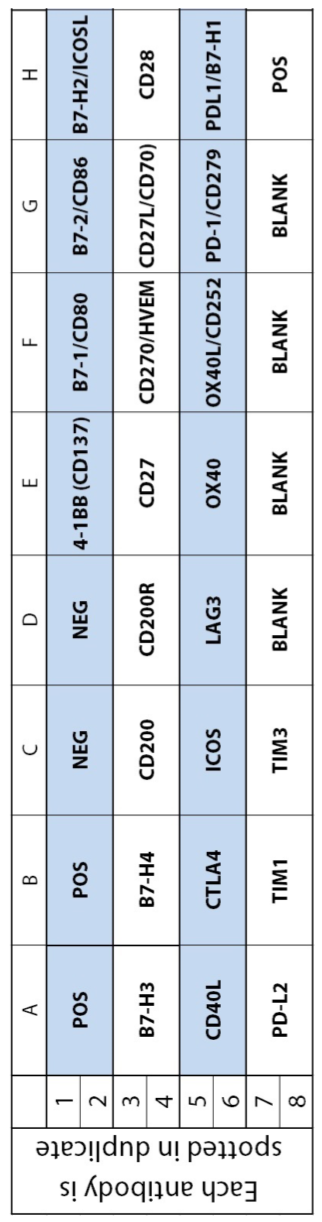


**Fig. S1 Molecule layout of immune checkpoint array spots co****rresponding to Figure 1B and 1C.**

Supplementary Figure 2


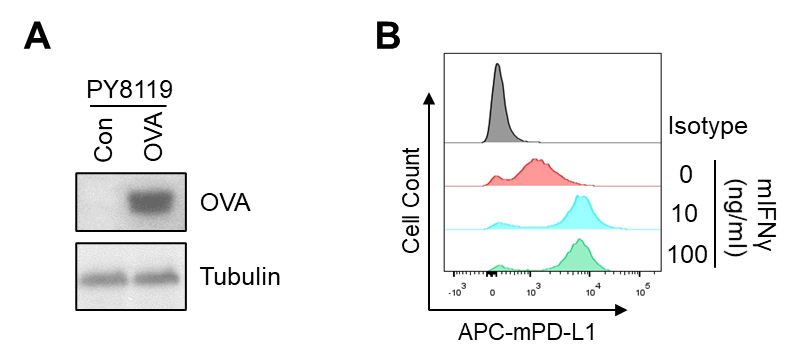


**Fig. S2 A** Immunoblot of OVA expression in wildtype and OVA-overexpression PY8119 cells. **B** Cell surface mPD-L1 expression of PY8119-OVA cells tested by flow cytometry in the absence or presence of mouse IFN-γ.

Supplementary Figure 3

**
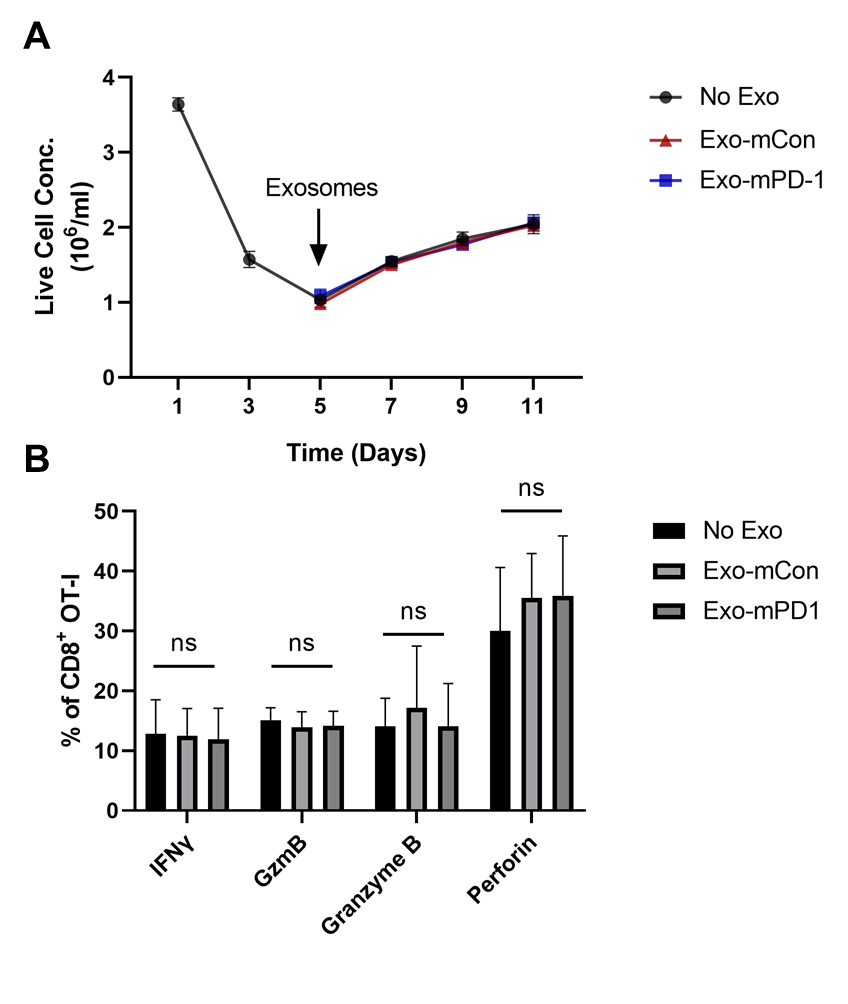
**

**Fig. S3 EL4-derived exosomes do not affect the proliferation and cytokine production of tumor-specific OT-I cells. A** Growth curve of exosome-treated OT-I cells (60μg per 1 × 10^6^ cells in 1 ml), exosomes-containing culture media were renewed every 2 days. **B** Flow cytometry analysis of intracellular IFN-γ, granzyme B, perforin and TNF-α in OT-I cells 6 days after exosome treatment (60μg per 1 × 10^6^ cells in 1 ml), exosomes-containing culture media were renewed every 2 days. Cells were pretreated by OVA_257-264_ peptide (2μg/ml) and Monensin Solution (BioLegend) for 8 h before staining.

Supplementary Figure 4

**
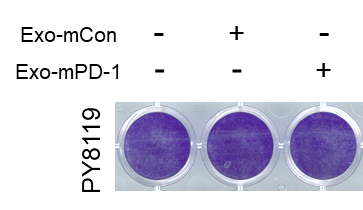
**

**Fig. S4 EL4-derived exosomes do not affect the growth and vitality of PY8119 cells.** 1.5 × 10^4^ PY8119-OVA cells were seeded into 24-well plates, and the cell vitality remained identical in 6 days in the presence of Exo-mCon or Exo-mPD-1. Exosome-containing culture media (30 μg/500 ul culture media/well) were renewed every 2 days.

Supplementary Figure 5


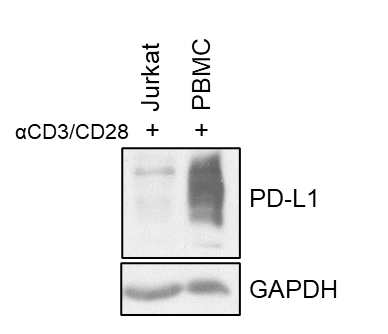


**Fig. S5** Immunoblot of PD-L1 expression in anti-CD3/CD28 antibody-activated (25 μl/ml) Jurkat-T and PBMC-T cells in 48 h.

Supplementary Table 1

| Cell line | Culture Media |
| --- | --- |
| Jurkat  Jurkat-PD-1-Myc  Jurkat-PD-1-EGFP | RPMI 1640 + 10% De-activated FBS  (56 °C for 30 min) |
| PBMCs | ImmunoCult™-XF T Cell Expansion Medium + 10% De-activated FBS + ImmunoCult™ Human T cell activator (25 μl/ml) + rhIL-2 (10 ng/ml) |
| EL4  EL4-shmPD-1 | DMEM + 10% De-activated FBS |
| OT-I | RPMI 1640 + 10% De-activated FBS + OVA_257-264_ peptide (2 μg/ml, InvivoGen) + rmIL-2 (10 ng/ml) + 50 μM 2-mercaptoethanol |
| MDA-MB-231  MDA-MB-231-PD-L1  BT-549  BT-549-PD-L1 | DMEM + 10% FBS |
| PY8119  PY8119-OVA | F-12K + 5% FBS |

**Table S1 Media applied for cell culture in this study.** All cells were incubated in a humidified atmosphere with 5% CO2 at 37 °C.

Supplementary Table 2

| Antibody | Manufacturer | Catalogue |
| --- | --- | --- |
| Human PD-1 | Cell Signaling Technology | #86163 |
| Human PD-L1 | Cell Signaling Technology | #13684 |
| Human PD-L1 extracellular domain | Cell Signaling Technology | #86744 |
| Mouse PD-1 | Cell Signaling Technology | #84651 |
| Human Calnexin | Cell Signaling Technology | #2679 |
| Alix | Cell Signaling Technology | #92880 |
| Erk-1/2 | Cell Signaling Technology | #4695 |
| Phospho-Erk1/2^Thr204/Tyr204^ | Cell Signaling Technology | #4370 |
| AKT | Cell Signaling Technology | #4691 |
| phosphor-AKT^Ser473^ | Cell Signaling Technology | #4051 |
| GAPDH | Cell Signaling Technology | #5174 |
| Alexa Fluo^TM^ 594 anti-rabbit IgG | Cell Signaling Technology | #8889 |
| Anti-rabbit IgG, HRP-linked | Cell Signaling Technology | #7074 |
| Anti-mouse IgG, HRP-linked | Cell Signaling Technology | #7076 |
| Mouse Calnexin | Santa Cruz Biotechnology | sc-46669 |
| Tsg101 | Santa Cruz Biotechnology | sc-7964 |
| CD9 | Santa Cruz Biotechnology | sc-13118 |
| CD63 | Santa Cruz Biotechnology | sc-365604 |
| Tubulin | Sigma-Aldric | T5168 |
| Alexa Fluor^TM^ 488 anti-human IgG Fc | BioLegend | 409321 |
| FITC anti-human CD274 (B7-H1, PD-L1) | BioLegend | 393605 |
| APC anti-human CD274 (B7-H1, PD-L1) | BioLegend | 374513 |

**Table S2 Antibodies applied in this study.**
